# NIR triggered PLGA coated Au-TiO_2_ core loaded CPT-11 nanoparticles for human papillary thyroid carcinoma therapy

**DOI:** 10.1080/10717544.2020.1775723

**Published:** 2020-06-09

**Authors:** Tianyu Yu, Lingling Tong, Yu Ao, Genmao Zhang, Yunpeng Liu, Hejia Zhang

**Affiliations:** a Department of Thyroid Surgery, China-Japan Union Hospital of Jilin University, Changchun, China; b Department of Obstetrics and Gynecology, China-Japan Union Hospital of Jilin University, Changchun, China; c Department of Pediatrics, The First Hospital of Jilin University, Changchun, China; d Department of Ultrasonography, China-Japan Union Hospital of Jilin University, Changchun, China; e Department of Thoracic Surgery, The First Hospital of Jilin University, Changchun, China

**Keywords:** Au@TiO_2_, CPT-11, thyroid cancer, anti-metastasis

## Abstract

MDR (multi-drug resistance) is one of the significant deterrents of effective chemotherapy for malignant growth. One of the powerful ways to deal with defeat of the MDR is to utilize inorganic nanoparticle-intervened tranquilize conveyance to build the medication aggregations in cancerous growth cells. In this work, we have developed the presentation that is accurately made of medication conveyance framework dependent on the TiO_2_ nanoparticles stacked CPT-11 to defeat the thyroid malignancy cells. The synthesized nanoparticles are characterized by spectroscopy methods (UV–vis, XPS, SEM, TEM, and DLS). The TEM results suggested that the shape of PLGA-Au-TiO_2_@CPT-11 of nanoparticles is ∼250 nm. After successful synthesis, we have evaluated the MTT of PLGA-Au-TiO_2_@CPT-11 nanoparticles with and without NIR radiations. Further, the morphological changes were observed using various biochemical stainings, such as acridine orange and ethidium bromide (AO–EB) and nuclear staining through Hoechst-33258. Also, migration and cell invasion were examined. The results show that these PLGA-Au-TiO_2_@CPT-11 and PLGA-Au-TiO_2_@CPT-11 + NIR nanoparticles exhibited promising antimetastatic property and reduced the cell invasion activity in B-CPAP and FTC-133 thyroid cancer cell lines. Based on the above findings, these PLGA-Au-TiO_2_@CPT-11 and PLGA-Au-TiO_2_@CPT-11 + NIR nanoparticles can be used as a promising candidate for the malignant thyroid cells.

## 1. Introduction

Though traditionally used cancer treatment such as chemotherapy and anti-cancer drugs has increased the survival rate and rehabilitation of cancer patients, it has some severe side effects. Anti-cancer drugs are tending to kill both cancer and non-cancerous cells (Asghar & Meyer, 2012; Li et al., 2014; Santha Moorthy et al., 2017). As of late, nanomaterial-based helpful strategies, for example, photograph warm treatment (PTT), photodynamic therapy (PDT), and controlled medication conveyance framework have gone to the spotlight. PTT evacuates harmful cells or tissue with the assistance of exterior light incited hyperthermia. It has negligible harm to healthy tissues of the beneficiary, and is helpful, non-obtrusive, remote-controllable and is protected (Shi et al., 2013; Bucharskaya et al., 2016; Kim et al., 2018). Au nanoparticles (AuNPs), copper NPs, carbon subordinates, change metal sulfides, dark titania (TiO_2_), and dark phosphorus (BPs) are a portion of the photothermal specialists their properties have been investigated seriously (Song et al., 2011; Gao et al., 2012; Ghosh et al., 2017; Yang et al., 2017; Rawal & Patel, 2019). Due to their high retention capacity in the close infrared locale (NIR) and phenomenal biocompatibility, AuNPs are increasingly favored among them. Be that as it may, to be great PTT operators for AuNPs, the retention of their confined surface plasmon reverberation (LSPR) must be altered to the area of 550–900 nm to permit the NIR laser treatment. This could be accomplished via cautiously observing AuNPs size and shape; however, it was hard to blend the material (Jana et al., 2001; Jana, 2005; Rao et al., 2017). Then again, the LSPR coupling was recommended as a novel methodology 15 among AuNPs and semiconductor. We find that the TiO_2_ shell altered with AuNPs is one of the best strategies for moving the assimilation of LSPR to a more drawn out wavelength. Inside the sub-wavelength areas contiguous with the AuNPs layer, the compacted free-space optical field permits significant electric field upgrade under full excitation, which improves the exhibition of photothermal transformation (Williams et al., 2008; Akhavan & Ghaderi, 2009; Lai et al., 2018; Kazimirova et al., 2019).

**Scheme 1.**
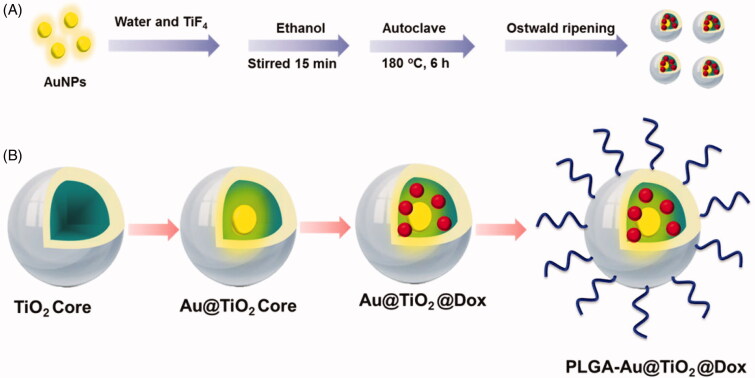
Graphic design of the preparation process of the (A) Au@TiO_2_ core and PLGA-Au-TiO_2_@CPT-11. (B) Schematic depiction of PLGA-Au-TiO2@CPT-11 with NIR irradiation showing potential chemotherapy effect.

CPT-11 (irinotecan) has been used to treat various types of cancer, such as leukemia, lung, breast, belly, vaginal sarcoma, and soft tissue sarcoma. CPT-11 intercalates between base pairs of DNA and locates the double helix in the lower groove (Chen et al., 2019; Fakiha et al., 2019; Fan et al., 2019; Si et al., 2019). CPT-11 intercalation blocks the development of the enzyme topoisomerase II, which winds DNA for transcription. To alleviate CPT-11 systemic toxicity due to its traditional delivery, it has been encapsulated in PEGylated liposomes to make it more targeted for tumor. Due to its central anthracycline chromophore group, CPT-11 is inherently fluorescent. CPT-11 accumulation in tissue or cells can, therefore, be observed in the visible range under fluorescence imaging systems (Kido et al., 2017; Hu et al., 2018; Kon et al., 2018; Liu & Liu, 2018). Nevertheless, absorption and interaction in emissions between CPT-11 and biological chromophores, such as hemoglobin, oxyhemoglobin, and melanin, hamper the efficacy of CPT-11 as an imaging agent for tumor detection and monitoring, especially *in vivo* (Bai et al., 2019; Liu et al., 2019; Malhotra et al., 2019; Ouyang et al., 2019; Song et al., 2019). This is the vital reason that other imaging devices, such as those with near-infrared (NIR) capability, should be used to track tumor location and CPT-11 treatment response. The present study documented a new, multifunctional CPT-11-loaded, PLGA-grafted Au@TiO_2_ core–shell NPs to PLGA-Au@TiO_2_@CPT-11 capable of selectively targeting and removing cancer cells by NIR-induced cancer therapy (Scheme 1(B)).

## 2. Experimental

The detailed synthetic procedures are depicted in Scheme 1(A).

### 2.1. Cell culture

Thyroid cancer cell lines B-CPAP and FTC-133 have been collected from the Department of Thoracic Surgery, the First Hospital of Jilin University, PR China. B-CPAP and NIH 3T3 cells are cultured in RPMI 1640 medium (Cell Signalling, Shanghai, China) and FTC-133 cancer cells are cultured in DMEM medium complemented by 10% fetal bovine serum (Cell Signalling, Shanghai, China), penicillin and 100 μg/mL streptomycin as antibiotics (Cell Signalling, Shanghai, China) in 96 well-grown were incubated under a humid atmosphere of 5% CO_2_ at 37 °C.

### 2.2. MTT assay

The MTT (3-(4,5-dimethylthiazol-2-yl)-2,5-diphenyltetrazolium bromide) test was conducted according to previously reported procedures. PLGA-Au-TiO_2_@CPT-11 and PLGA-Au-TiO_2_@CPT-11 + NIR in a concentration range of 0–25 μg/mL soluble in DMSO were applied to wells 24 h after 3 × 10^3^ cells per well in a fresh culture medium of 200 μL. The DMSO was used as vehicle control. The percentage of cell inhibition by gold nanoparticles was calculated and plotted in the graph by using GraphPad Prism software (La Jolla, CA).

### 2.3. Dual (AO–EB) staining

Apoptotic morphological changes upon treatment with IC_50_ concentration of PLGA-Au-TiO_2_@CPT-11 and PLGA-Au-TiO_2_@CPT-11 + NIR nanoparticles against cancer B-CPAP and FTC-133 cell lines were measured by using acridine orange and ethidium bromide (AO/EB) and Hoechst 33344 staining. After staining, the cells were visualized under a fluorescence microscope (Accu Scope EXI-310) at ×20 magnification (Subarkhan & Ramesh, 2016; Mohamed Kasim et al., 2018).

### 2.4. Wound healing assay

After 24 h at 37 °C, B-CPAP and FTC-133 cells are extended to 90% juncture in a six-well dish. A scratch over the cell monolayer was delivered utilizing a clean 10 μL pipette tip. Following treatment with PLGA-Au-TiO_2_@CPT-11 and PLGA-Au-TiO_2_@CPT-11 + NIR (IC_50_ concentration, individually) and 0.1% DMSO as control cells, wound pictures are gathered by optical microscopy at time0 in the wake of scratching and toward the finish of a 24-hour brooding period. To quantify the movement rate, the separation between the first twisted and the separation between the mending wound and the pipet tip at 24 h after scratching was thought about (Mohamed Subarkhan et al., 2019). The wound area was examined through the Image J software (Bethesda, MD).

### 2.5. Cell invasion assay

Matrigel framework (BD Biosciences, Franklin Lakes, NJ) was placed in Transwell channels (30 μL/well, 8.0 μm PET, Millipore, Billerica, MA) and permitted to finish gelling for 1 h at 37 °C. A limit of 200 μL of RPMI-1640 and DMEM medium containing 4 × 10^4^ B-CPAP and FTC-133 cells were added to the upper chambers and 700 μL of RPMI-1640 medium with 10% FBS was added to the lower chambers. In this way, the wells in the upper chambers are taken care of with PLGA-Au-TiO_2_@CPT-11 and PLGA-Au-TiO_2_@CPT-11 + NIR (IC_50_ concentration, individually) at 37 °C for 24 h. Following 24 h of brooding, cotton swabs were utilized to scratch the Matrigel and the cells that stayed in the upper chambers. Next, the B-CPAP and FTC-133 cells on the base surface of the layer were fixed with methanol for 10 min and recolored with 0.5% purple precious stone for 15 min. Intrusive cells on the film were washed with distilled water and shot under an optical magnifying lens. Cells have been included in any event three irregular infinitesimal fields (amplification ×200). The examinations were rehashed multiple times (Gialeli et al., 2011; Zou et al., 2016; Mahkamova et al., 2019). The invaded cells were examined through the Image J software (Bethesda, MD).

## 3. Results and discussion

### 3.1. Synthesis and structural illustration of PLGA-Au-TiO_2_@CPT-11

The PLGA-Au-TiO_2_@CPT-11 and PLGA-Au-TiO_2_@CPT-11 + NIR were prepared with some alteration in compliance with previous methods. Scheme 1(A) displays the overall synthetic process.

**Figure 1.**
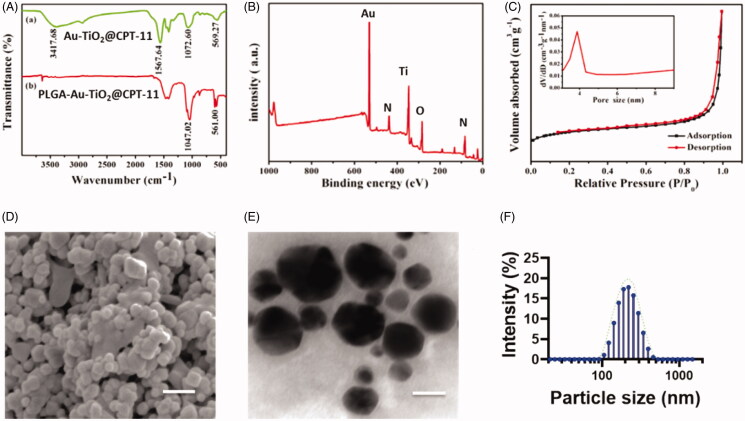
Physicochemical characteristics of functionalized Au-TiO_2_@CPT-11 and PLGA-Au-TiO_2_@CPT-11. (A) IR spectrum of Au-TiO_2_@CPT-11 and PLGA-Au-TiO_2_@CPT-11. (B) XPS analysis of PLGA-Au-TiO_2_@CPT-11. (C) N2 adsorption/desorption isotherms. (D) SEM image of PLGA-Au-TiO_2_@CPT-11. Scale bar 200 nm. (E) TEM images and size distributions and the scale bar 200 nm. (F) DLS of the size distribution of PLGA-Au-TiO_2_@CPT-11.

The infrared spectroscopy investigations are used to evaluate the coordination modes of the nanoparticles (Figure 1(A)). The UV–vis displays the significant peaks, that show 3417.68 and 1567 C=O, and C=N frequencies are indicating the formation of conjugate of the PLGA. The free PLGA-Au-TiO_2_@CPT-11 does not show the transmittance in UV–vis spectroscopy. These results clearly indicate the formation of the PLGA-Au-TiO_2_@CPT-11. The elemental mapping analysis is used to describe PLGA-Au-TiO_2_@CPT-11 functionalization. Figure 1(B) displays the unvarying composition of elements (Au, Ti, and O). The mapping of Ti elements was based on the TiO_2_ surface and the mapping of Au elements, indicating the presence of AuNPs. The nitrogen physicochemical properties were also examined by the PLGA-Au-TiO_2_@CPT-11. Figure 1(C) shows the N_2_ adsorption and desorption isotherms and core size of the samples. The PLGA-Au-TiO_2_@CPT-11 shows well defined hysteresis loop, displays well-built appearances. The average diameter displays average diameter pore size 9.02 nm, indicates that active sites and passage for CPT-11 into the core–shell. PLGA-Au-TiO2@CPT-11 core–shell NPs have been studied by electron microscopy SEM and TEM transmission. The SEM image is shown in Figure 1(D). It shows large core volumes and high specific surface areas, which absorb a large amount of CPT-11 accumulations. Figure 1(E) displays descriptions of PLGA-Au-TiO_2_@CPT-11 with high-resolution TEM (HR-TEM). The darker spots were the Au core due to the disparity masses of Au and Ti, while the lighter area was shells of TiO_2_. It should be remembered that Au-NPs were enclosed by a layer of TiO_2_ surface. In fact, the resonating gap in each nanohybrid between the center and the core might be undoubtedly noticed. The PLGA-Au-TiO_2_@CPT-11 NPs displayed a unvarying 200 nm width. The findings of dynamic light scattering (DLS) showed that PLGA-Au-TiO_2_@CPT-11 core–shell nanoparticles average hydrodynamic particle size was 233.5 ± 69.3 (Figure 1(F)), showing highly stable in DD water without forming any aggregation. An HR-TEM photo showed the polycrystalline core of TiO_2_ with numerous minor and arbitrarily focused crystallization (Shen et al., 2011; Llinàs et al., 2018; Tambe et al., 2018).

### 3.2. *In vitro* cytotoxicity

**Figure 2.**
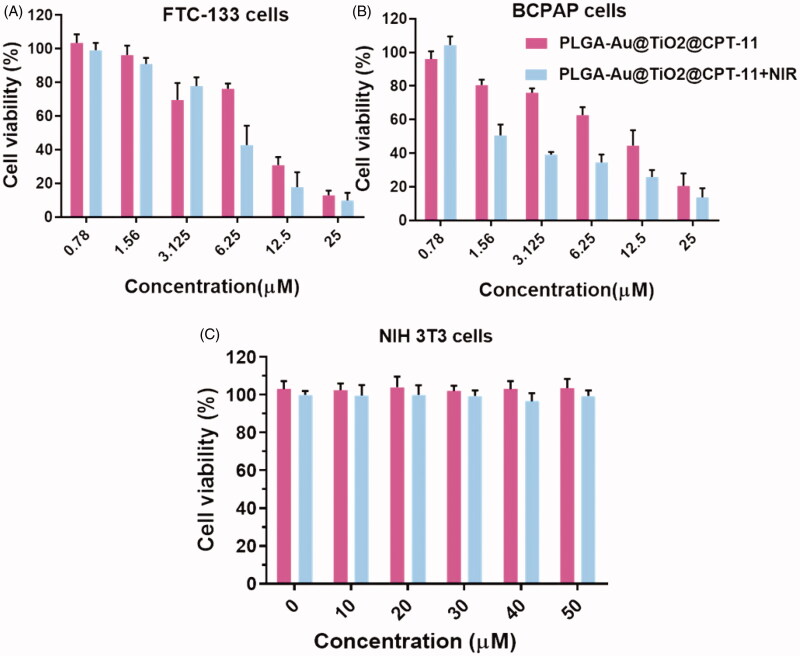
*In vitro* cytotoxicity of B-CPAP and FTC-133 thyroid cancer cell lines with NIH3T3 non-cancerous cell lines.

The MTT assay was used to make a preliminary assessment of the growth-inhibiting action of PLGA-Au-TiO_2_@CPT-11 and PLGA-Au-TiO_2_@CPT-11 + NIR in B-CPAP and FTC-133 thyroid cancer cell lines to determine the respective IC_50_ values at the individual time points (Yu et al., 2016; Kim et al., 2018; Mahkamova et al., 2019). With varying concentrations of PLGA-Au-TiO_2_@CPT-11 and PLGA-Au-TiO_2_@CPT-11 + NIR, the cells have been treated for 24 hours. It exhibited a significant dose-dependent inhibition of growth. As shown in Figure 2, PLGA-Au-TiO_2_@CPT-11 and PLGA-Au-TiO_2_@CPT-11 + NIR induced a marked decrease in cell growth with IC_50_ values of 8.98 and 4.90 µM for B-CPAP cells and 8.52 and 2.31 µM for FTC-133 cells at 24 h, respectively. Hence, they treated PLGA-Au-TiO_2_@CPT-11 and PLGA-Au-TiO_2_@CPT-11 + NIR cells at the IC_50_ concentration in all subsequent studies, unless otherwise specified. Further, we examined the non-cancerous NIH-3T3 cells with the fabricated nanoparticles, PLGA-Au-TiO_2_@CPT-11 and PLGA-Au-TiO_2_@CPT-11 + NIR exhibited the high cytotoxicity in NIH3T3 cells (Figure 2). These results thus evidenced the advantages of the PLGA-Au-TiO_2_@CPT-11 and PLGA-Au-TiO_2_@CPT-11 + NIR as cancer-selective agents, and they should have the potential to reduce the toxicity to healthy cells and organs when considering the *in vivo* use.

### 3.3. Morphological assessment

**Figure 3.**
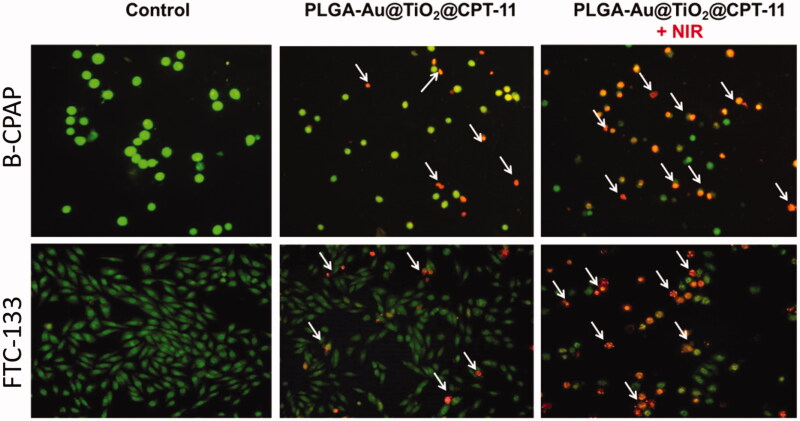
Dual AO/EB fluorescent staining of B-CPAP and FTC-133 thyroid cancer cell lines after treatment with PLGA-Au-TiO_2_@CPT-11 and PLGA-Au-TiO_2_@CPT-11 + NIR (IC_50_ concentration) for 24 h.

By using a fluorescent microscopic analysis of the AO–EB and Hoechst33258-stained in B-CPAP and FTC-133 thyroid cancer cell lines, characteristic morphological changes induced by PLGA-Au-TiO_2_@CPT-11 and PLGA-Au-TiO_2_@CPT-11 + NIR were evaluated (Figure 3). The nanoparticle induces cell death via two pathways, such as apoptosis and necrosis, after treatment with their IC_50_ meditations at 24 hours. Ironically, PLGA-Au-TiO_2_@CPT-11 + NIR shows a higher percentage of the apoptotic mode of cell death than PLGA-Au-TiO_2_@CPT-11.

**Figure 4.**
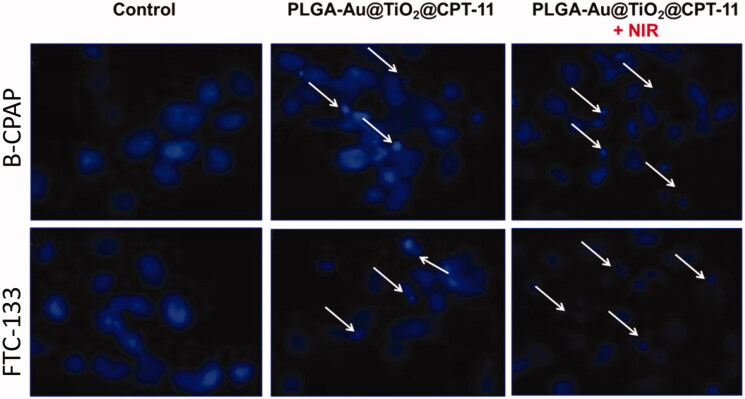
Nuclear staining of B-CPAP and FTC-133 thyroid cancer cell lines after treatment with PLGA-Au-TiO_2_@CPT-11 and PLGA-Au-TiO_2_@CPT-11 + NIR (IC_50_ concentration) for 24 h.

Once Hoechst-33258 staining is used, chromatin fragmentation, bi- and/or multinucleation, cytoplasmic vacuolation, nuclear swelling, cytoplasmic bleating, and late apoptosis suggestion of dot-like chromatin condensation are detected on thyroid cancer cells morphological change (Kasibhatla et al., 2006; Pang et al., 2013; Liu et al., 2015; Mohamed Subarkhan et al., 2016). The observed cytological changes are secreted into four sorts rendering to the emission of fluorescence and the morphological characteristics of chromatin condensation in the AO–EB stained nucleus: (i) live cells with consistently green fluorescent nucleus with an extremely structured structure; (ii) early apoptotic cells (which still have intact membranes but have begun to undergo DNA fragmentation) with green fluorescent nodes, but peri-nuclear chromatin nuclear chromatin condensation is visible as bright green patches or fragments; (iii) late apoptotic cells with orange to red fluorescent nodes with condensed or fragmented chromatin; (iv) necrotic cells swollen to enormous sizes, with uniformly orange to the red fluorescent nucleus with no sign of chromatin fragmentation. All these morphological modifications suggest that the thyroid cancer cells undergo apoptosis mode of cell death over the treatment of 24 hours (Figure 4). It has been stated that the anti-cancer action of certain PLGA-Au-TiO_2_@CPT-11 and PLGA-Au-TiO_2_@CPT-11 + NIR depends on their interaction with different proteins and their modes of binding to duplex DNA, full binding of plasma proteins may result in drastic alterations or even loss of the biological properties of PLGA-Au-TiO_2_@CPT-11 and PLGA-Au-TiO_2_@CPT-11 + NIR nanoparticles.

### 3.4. Migration and cell invasion assay

**Figure 5.**
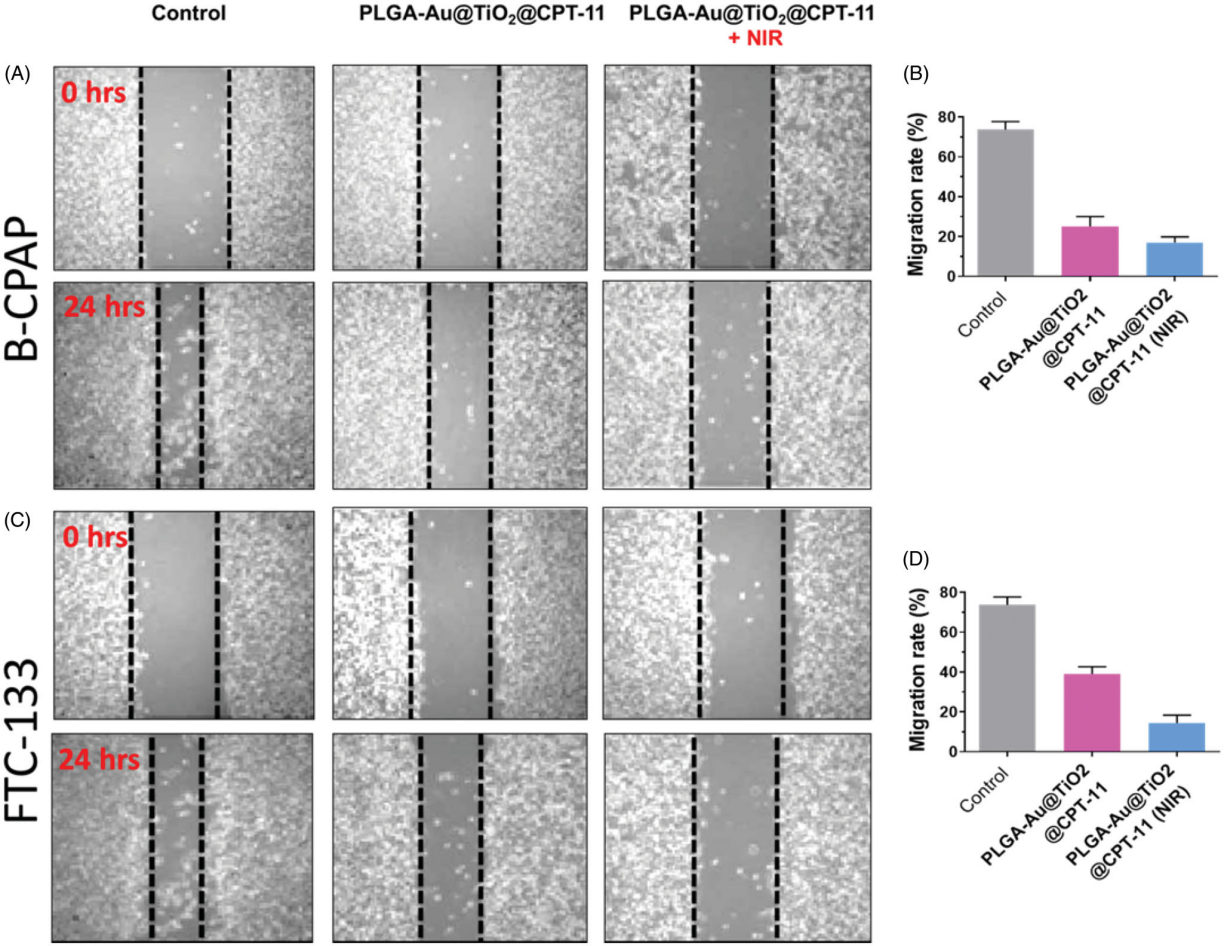
(A, C) The migration of B-CPAP and FTC-133 thyroid cancer cell lines was detected by a scratch assay. The B-CPAP and FTC-133 cells were treated with PLGA-Au-TiO_2_@CPT-11 and PLGA-Au-TiO_2_@CPT-11 + NIR (IC_50_ concentration) for 24 h. (B, D) Quantification analysis showed that PLGA-Au-TiO_2_@CPT-11 and PLGA-Au-TiO_2_@CPT-11 + NIR reduced B-CPAP and FTC-133 migration.

Metastasis and invasion are significant occurrences in the future period of cancer development. For successful cancer treatment, metastatic suppression and invasion are essential (Jin et al., 2012; Zhu et al., 2016; Guo et al., 2017). Cell proliferation happens through biological procedures, which play a vital role in the development of numerous sicknesses, including thyroid cancer. *In vitro* migration assays are necessary for understanding the mechanism of cell migration and for recognizing the inhibitory potential of PLGA-Au-TiO_2_@CPT-11 and PLGA-Au-TiO_2_@CPT-11 + NIR at IC_50_ concentration in B-CPAP and FTC-133 thyroid cancer cell lines (Figure 5).

**Figure 6.**
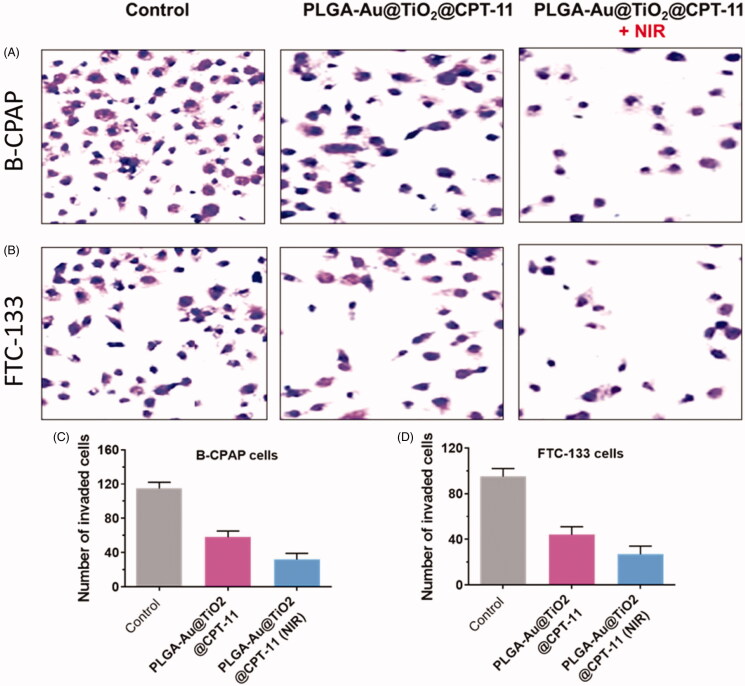
(A, B) The invasion of B-CPAP and FTC-133 thyroid cancer cells was detected via transwell techniques. B-CPAP and FTC-133 cells were treated with PLGA-Au-TiO_2_@CPT-11 and PLGA-Au-TiO_2_@CPT-11 + NIR for 24 h. (C, D) The number of invading thyroid cancer cells upon treatment with nanoparticles was remarkably decreased compared with that of the control cells.

In B-CPAP and FTC-133 thyroid cancer cell lines, migration after treatment with PLGA-Au-TiO_2_@CPT-11 and PLGA-Au-TiO_2_@CPT-11 + NIR decreased significantly to IC_50_ concentration which was substantially less active. For instance, the wound closure ratios are 18.8%, 23.3%, and 42.3% respectively for PLGA-Au-TiO_2_@CPT-11 and PLGA-Au-TiO_2_@CPT-11 + NIR. Furthermore, during cancer development, active anti-cancer medications must be able to prevent the migration of cancer cells from main places to other tissues in malignancy candidates. Hence, the transwell assay then tested the invasiveness of cancer cells (Forner et al., 2012; Jin et al., 2012; Ni et al., 2017). Invasive B-CPAP and FTC-133 cells are sowed on matrigel coated tissue and preserved for 24 h with PLGA-Au-TiO_2_@CPT-11 and PLGA-Au-TiO_2_@CPT-11 + NIR (IC_50_ concentration, respectively). During therapy with PLGA-Au-TiO_2_@CPT-11 and PLGA-Au-TiO_2_@CPT-11 + NIR, the number of invading cells decreased significantly relative to untreated cells (Figure 6). Based on these findings, delivery indicate that, in addition to *in vitro* cytotoxic activity, these PLGA-Au-TiO_2_@CPT-11 and PLGA-Au-TiO_2_@CPT-11 + NIR nanoparticles can suppress metastases and the invasion of B-CPAP and FTC-133 thyroid cancer cells.

## 4. Conclusions

In conclusion, we have successfully designed and demonstrated the Au@TiO_2_ loaded with CPT-11 for the treatment of thyroid cancer cells. The results of PLGA-Au-TiO_2_@CPT-11 nanoparticles were characterized through the various spectroscopy methods (UV–vis, XPS, SEM, TEM, and DLS). The SEM and TEM results suggested that the shapes of PLGA-Au-TiO_2_@CPT-11 nanoparticles are well organized and shaped. After positive synthesis, we have evaluated the MTT of PLGA-Au-TiO_2_@CPT-11 nanoparticles with and without NIR radiations. Further, the morphological changes were observed using AO–EB and Hoechst-33258 staining. The results show these PLGA-Au-TiO_2_@CPT-11 and PLGA-Au-TiO_2_@CPT-11 + NIR nanoparticles exhibited promising antimetastatic property and reduced the cell invasion activity in FTC-133 and B-CPAP thyroid cancer cell lines. The findings of PLGA-Au-TiO_2_@CPT-11 and PLGA-Au-TiO_2_@CPT-11 + NIR nanoparticles would invent the probable use in thyroid patents with improved therapeutic and future clinical investigation.
